# Clinical significance of serum synaptophysin-like 1 protein levels in breast cancer

**DOI:** 10.5937/jomb0-46198

**Published:** 2024-04-23

**Authors:** Yagmur Ozge Turac Kosem, Hafize Uzun, Mehmet Velidedeoglu, Pınar Kocael, Seyma Dumur, Osman Simsek

**Affiliations:** 1 Istanbul University-Cerrahpas, Cerrahpa a Faculty of Medicine, Department of General Surgery, Istanbul, Turkey; 2 Istanbul Atlas University, Faculty of Medicine, Department of Medical Biochemistry, Istanbul, Turkey

**Keywords:** synaptophysin-like 1, breast cancer, dense breasts, tumor markers, sinaptofizin-sličan 1, rak dojke, gustoća dojki, tumorski markeri

## Abstract

**Background:**

Mammography, used for breast cancer (BC) screening, has limitations such as decreased sensitivity in dense breasts. Currently used tumor markers are insufficient in diagnosing breast cancer. In this study, we aimed to investigate the relationship between serum levels of synaptophysin-like protein 1 (SYPL1) and BC and compare SYPL1 with other blood tumor markers.

**Methods:**

The study group consisted of 80 female patients with a histopathological diagnosis of invasive BC who received no radiotherapy/chemotherapy. The control group was 72 women with no previous history of breast disease and evaluated as Breast Imaging Reporting and Data Systems (BI-RADS 1-2) on imaging. Serum SYPL1, cancer antigen 15-3 (CA 15-3), and carcinoembryonic antigen (CEA) were measured in both groups.

## Abstract (sr)

**Uvod:**

Mamografija, koja se koristi za skrining raka dojke (RD), ima ograničenja poput smanjene osetljivosti kod veće gustoće dojki. Tumorski markeri koji se trenutno koriste nisu dovoljni za dijagnostikovanje raka dojke. Cilj ove studije je bio da seistrži odnos između nivoa serumskog proteina SYPL1 i raka dojke (RD), i da se uporedi SYPL1 sa drugim tumorskim markerima u krvi.

**Metode:**

Studijska grupa je obuhvatila 80 pacijentkinja sa histopatološkom dijagnozom invazivnog raka dojke koje nisu primale radioterapiju/hemoterapiju. Kontrolna grupa je obuhvatila 72 žene bez prethodne anamneze bolesti dojke koje su ocenjene kao BI-RADS 1-2 (Breast Imaging Reporting and Data Systems). Mereni su nivoi SYPL1, anti-gena raka 15-3 (CA 15-3) i karcinoembriogenog antigena (CEA) u serumu u obe grupe.

**Rezultati:**

Dijagnostičke vrednosti proteina SYPL1, CEA i CA15-3 u dijagnostikovanju raka dojke su bile statistički značajne. Osetljivost SYPL1 je iznosila 48,75%, uz specificitet od 80,56%. CA15-3 je imao osetljivost od 80% i specificitet od 49,30%. Nije bilo statistički značajne korelacije između nivoa seruma SYPL1 i dijametra tumora, metastaza u limfnim čvorovima, metastaza na udaljenim organima i stadijuma.

**Zaključak:**

Serumski SYPL1održvao je veću sposobnost diskriminacije za RD. Nivo seruma SYPL1 se može koristiti sa visokim specificitetom u dijagnostikovanju RD. Međutim, SYPL1 ima nisku dijagnostičku vrednost sam za sebe.

## Introduction

Breast cancer (BC) is the most common type of cancer in the world after lung cancer. There are many pathological variants. Invasive ductal carcinoma is seen in 80% of cases [1]. Being a woman is the most critical risk factor for BC. Apart from this, advanced age (>50 years), early menarche (<12 years), late menopause (>55 years), never giving birth, not breastfeeding, giving birth at an advanced age (>35 years), applied to the thorax in childhood radiotherapy, long-term postmenopausal hormone replacement therapy, chronic alcohol use, postmenopausal obesity, benign proliferative breast diseases, presence of atypical hyperplasia as a result of previous biopsy, presence of lobular carcinoma in situ, dense breast structure on mammography, familial and genetic factors increase the risk of BC [2] [3] [4] [5]. The most common genetic risk factors are BRCA1 and BRCA2 mutations [6].

The most commonly used marker for BC is CA 15-3 elevation. This marker is primarily used to evaluate the response to treatment in metastatic BC and to follow up on recurrence. It is not used in BC screening because of its low sensitivity. CA 15-3 may also be elevated in other cancers, such as lung, large intestine, pancreatic, liver, ovarian, cervical, and uterine wall cancers, apart from BC. The currently accepted gold standard method for BC screening is mammography [7].

There are many newly studied blood biomarkers. Synaptophysin-like 1 (SYPL1) belongs to the SYP (synaptophysin) family known as SYPL or pantophysin. It is a vesicle membrane protein found in neuronal and non-neuronal tissues. Malignant melanomas, urothelial cancers, and a few cases of gastric adenocarcinoma showed strong cytoplasmic positivity [8] [9]. Malignant gliomas, breast, prostate, colorectal, testicular, and skin cancers, weak staining, or negative cells are seen in a few cases [9] [10] [11] [12] [13]. SYPL1 has prognostic value with a high expression favorable in renal cancer [14].

Due to the limitations of mammography for BC, which is such a major public health problem, it is observed that there is a need for new tests that can accompany mammography and be used in screening. Therefore, our study aimed to determine the screening value of serum SYPL1 protein as a blood biomarker in BC.

## Materials and methods

All subjects who participated in the study gave informed consent, and the study was approved by the ethics committee of Istanbul University-Cerrahpasa, Cerrahpas Medical Faculty (approval number 12/10/2020, 133998). This study was conducted following the Declaration of Helsinki.

A total of 80 patients with breast mass who had breast surgery were enrolled consecutively between December 2020 and October 2021 in the Istanbul University-Cerrahpasa, Cerrahpa a Medical Faculty. The study group consisted of 80 female patients (age: 51.74±11.77) with a histopathological diagnosis of invasive breast carcinoma and didn’t receive any radiotherapy/chemotherapy. The control group consisted of 72 healty women (age: 46.22±11.91) with no previous history of breast disease and evaluated as BIRADS1-2 on imaging.

### Inclusion criteria

Invasive BC compatible results in breast tru-cut biopsy results of those who will form the study group with the disease;

being over the age of 18 and being female;

not having received any chemotherapy or radiotherapy treatment;

individuals in the control group do not have any history of breast disease, are in the Breast Imaging Reporting and Data Systems (BI-RADS 1-2) category in routine screening imaging;

having read, understood, and approved the informed consent form.

### Exclusion criteria

No diagnosis of pathologically invasive BC was found in the people who will be included in the study group;

having received chemotherapy or radiotherapy;

any history of breast disease in the individuals in the control group; Having been diagnosed with another system cancer before;

volunteers who did not sign the consent form;

serum samples were obtained after at least 30 min of clotting by centrifugation at 2,500 g for 15 min and were stored at -80°C until assayed for determination of SYLP1 and biochemical parameters.

### Serum synaptophysin-like 1 (SYPL1) analysis

Serum SYLP1 levels were measured using the Human SYLP1 ELISA Kit (abbexa, Cat. no:abx548201, Houston, USA). The intra and inter-assay variation coefficients were 5.7% (n=20) and 7.2% (n=20), respectively.

Tumor markers were measured using an IMMU-LITE 2000 (DPC, Los Angeles, CA).

Biochemical parameters were measured using the spectrophotometric method by the autoanalyzer (Hitachi Modular System, Roche Diagnostic, Corporation, Hague Road, Indianapolis, IN). CRP values were measured with the turbidimetric method by auto analyzer (ADVIA 1800 Auto Analyzer, Siemens medical Sol., Deerfield, IL).

### Statıstıcal analysıs

Statistical analyses were performed using SPSS 22.0 (SPSS Inc., Chicago, IL) and Medcalc 14.8.1package programs. Whether the quantitative variables were suitable for normal distribution was examined with the Kolmogorov-Smirnov test. Independent groups were compared for normally distributed variables with independent samples t-test or one-way analysis of variance (ANOVA) and for non-normally distributed variables with Mann Whitney U or Kruskal Wallis H test. Pearson or Spearman correlation analysis was used to examine the relationship between quantitative variables. The diagnostic values of SYPL1, CEA, and CA15-3 variables in diagnosing BC were determined by ROC analysis. Descriptive statistics of quantitative variables conforming to normal distribution were shown as mean ± standard deviation, and descriptive statistics of non-normally distributed quantitative variables were shown as median (25-75th percentile). Descriptive statistics for qualitative variables were expressed as frequency (%). p<0.05 values were considered statistically significant.

## Results

The mean age of the patient group (n=80, 51.74±11.77 years) was statistically significantly higherthan the mean age of the group consisting of healthy individuals (n=72, 46.22±11.91 years) (p=0.005). It was found that there was a weak positive correlation between creatinine and LDH levels and SYPL1 level (r=0.175, p=0.031; r=0.169, p=0.042, respectively). Glucose, AST, and ALT levels were not associated with SYPL1 (p>0.05).

The qualitative and quantitative variables of the patients are given in Table 1.

**Table 1:** Descriptive statistics on qualitative and quantitative variables. ER, estrogen receptor; PR, progesterone receptor

Tumor type	Invasive ductal carcinoma<br>Invasive lobular carcinoma<br>Other	69 (86.3)<br>4 (5)<br>7 (8.8)
Breast type	A<br>B<br>C<br>D	10 (9.1)<br>32 (29.1)<br>52 (47.3)<br>16 (14.5)
BI-RADS	Category 0<br>Category 1<br>Category 2<br>Category 3<br>Category 4<br>Category 5<br>Category 6	11 (7.3)<br>2 (1.3)<br>67 (44.7)<br>1 (0.7)<br>25 (16.7)<br>40 (26.7)<br>4 (2.7)
Stage	Stage 1A<br>Stage 2A<br>Stage 2B<br>Stage 3A<br>Stage 3B<br>Stage 3C<br>Stage 4	20 (25.6)<br>28 (35.9)<br>15 (19.2)<br>4 (5.1)<br>1 (1.3)<br>1 (1.3)<br>9 (11.5)
Hormone<br>type	Luminal<br>Luminal B HER2 (-)<br>Luminal B HER2 (+)<br>Her2 (+)<br>Triple negative	30 (39)<br>22 (28.6)<br>13 (16.9)<br>6 (7.8)<br>6 (7.8)
Grade	Grade 1<br>Grade 2<br>Grade 3	4 (5.1)<br>39 (49.4)<br>36 (45.6)
ER	Negative<br>Positive	14 (17.9)<br>64 (82.1)
PR	Negative<br>Positive	15 (19.2)<br>63 (80.8)
HER-2/neu<br>(cerB-2)<br>amplification	Negative<br>Positive	62 (80.5)<br>15 (19.5)
Ki-67	0–15<br>16–35<br>>35	33 (42.3)<br>21 (26.9)<br>24 (30.8)

There was no significant relationship between age, tumor diameter variables, and SYPL1 (p>0.05).However, there was a weak positive correlation between BI-RADS and SYPL1 (r=0.165; p=0.043). No difference was found between SYPL1 protein levels and subgroups of breast type, lymph node biopsy in the axilla, TNM stage, prognostic stage, hormone type, grade, ER, PR, c-erB-2, Ki-67, multifocal tumor variables (Table 2).

**Table 2:** Descriptive statistics and comparison results of SYPL1 protein in terms of breast type, axillary lymph node biopsy, T, N, M, stage, prognostic stage, hormone type, grade, ER, PR, CerbB2 amplification, Ki67, and subgroups of multifocal tumors. a: Independent samples t-test<b<br>>c: Kruskal-Wallis H test<br>d: Mann-Whitney U teste: ANOVA<br>Descriptive statistics are shown as mean±standard deviation or median (25th–75th percentile)<br>χ^2^, F, U, t: Test statistic

GRUPLAR	SYPL1	χ^2^ / F / U / t	P
Breast type<br>A (n=10)<br>B (n=32)<br>C (n=52)<br>D (n=16)	<br>13.99±12.71<br>10.23±3.71<br>9.60±5.58<br>12.21±5.08	1.952	0.126^c^
Axillary lymph node biopsy<br>Benign (n=17)<br>Malign (n=31)	<br>12.66 (9.42–17.20)<br>10.43 (6.36–13.65)	184.00	0.087^d^
T<br><20 mm (n=25)<br>≥20 mm (n=52)	<br>11.67 (9.04–15.63)<br>11.92 (6.71–15.07)	610.00	0.663^d^
N<br>N0 (n=45)<br>N1 (n=23)<br>N2 (n=6)<br>N3 (n=4)	<br>12.66 (8.47–15.76)<br>1.85 (1.40–2.63)<br>1.85 (1.04–5.86)<br>3.20 (1.27–4.27)	2.030	0.566^c^
M<br>Distant metastasis (-) (n=67)<br>Distant metastasis (+) (n=11)	<br>12.6 (6.92–14.88<br>12.66 (8.33–16.62)	431.00	0.370^d^
Stage<br>Early stage (n=48)<br>Locally advanced and metastatic stage (n=30)	<br>12.09±5.20<br>11.85±8.07	0.160	0.873^a^
Prognostic stage <br>1A (n=19) <br>1B (n=15) <br>2A (n=19) <br>2B (n=9) <br>3A (n=3) <br>3B (n=3) <br>3C (n=1) <br>4 (n=9)	<br>12.26 (8.89–15.87)<br>12.41 (5.51–17.36)<br>13.15 (10.68–15.13)<br>10.18 (6.08–13.10)<br>13.15 (6.92–6.92)<br>9.17 (7.76–7.76)<br>4.67 (4.67–4.67)<br>11.67 (8.05–17.73)	6.305	0.505^c^
Hormone Type <br>Luminal A (n=30) <br>Luminal B HER2 (-) (n=22) <br>Luminal B HER2 (+) (n=13) <br>HER2 (+) (n=6) <br>Triple negative (n=6)	<br>12.66 (7.90–15.45)<br>13.15 (8.12–17.12)<br>7.76 (5.80–16.25)<br>10.68 (5.44–13.19)<br>10.38 (8.05–12.82)	3.978	0.409^c^
Grade <br>Grade 2 (n=39) <br>Grade 3 (n=36)	<br>12.26 (6.64–15.38)<br>12.11 (8.11–15.63)	740.50	0.683^d^
ER <br>Negative (n=14) <br>Positive (n=64)	<br>10.51 (8.47–13.94)<br>12.54 (7.48–15.64)	505.00	0.458^d^
PR <br>Negative (n=15) <br>Positive (n=63)	<br>10.58 (6.92–14.79)<br>12.41 (7.48–15.38)	503.00	0.699^d^
Cerb2 Amplification <br>Negative (n=62) <br>Positive (n=15)	<br>12.60 (7.90–15.44)<br>10.18 (5.80–14.79)	384.00	0.297^d^
Ki67 <br>0-15 (n=33) <br>16-35 (n=21) <br>>35 (n=24)	<br>11.27±5.14<br>11.79±5.26<br>11.98±6.43	0.578	0.563^e^
Multifocal tumor <br>Unifocal tumor (n=53) <br>Multifocal tumor (n=15)	<br>12.41 (9.18–15.76)<br>7.76 (5.80–13.15)	271.50	0.062^d^

Descriptive statistics and comparison results of CA15-3 protein in M (metastasis), stage, prognostic stage, hormone type, and multifocal tumor subgroups are given in Table 2. Table 3

**Table 3:** Descriptive statistics and comparison results of CA15-3 protein in M (metastasis), stage, prognostic stage, hormone type, and multifocal tumor subgroups. c: Kruskal-Wallis H test<br>d: Mann-Whitney U test<br>*: 1B-4 are different from each other.<br>**: Luminal a - Luminal b her2+ are different from each other<br>Descriptive statistics median (shown as 25th–75th percentile)<br>χ^2^/ U: Test statistic

Gruops	CA15-3	χ^2^ / U	P
*M*<br>Distant metastasis (-) (n=67)<br>Distant metastasis (+) (n=11)	<br>17.78 (13.94–24.27)<br>27.30 (19.10–70.10)	566.00	0.005^d^
*Stage*<br>Early stage (n=48)<br>Locally advanced and metastatic stage (n=30)	<br>17.67 (13.23–22.91)<br>21.85 (16.34–38.41)	982.00	0.007^d^
*Prognostic stage*<br>1A (n=19)<br>1B (n=15)<br>2A (n=19)<br>2B (n=9)<br>3A (n=3)<br>3B (n=3)<br>3C (n=1)<br>4 (n=9)	<br>19.86 (17.19–28.77)<br>14.01 (8.02–24.05)*<br>19.10 (13.47–21.76)<br>18.60 (15.35–22.20)<br>21.32 (13.98–13.98)<br>14.77 (8.91–8.91)<br>34.81 (34.81–34.81)<br>40.68 (18.88–74.85)	14.696	0.004^c^
*Hormone Type*<br>Luminal A (n=30)<br>Luminal B Her2 (-) (n=22)<br>Luminal B Her2 (+) (n=13)<br>Her2 (+) (n=6)<br>Triple negatif (n=6)	<br>17.15 (8.83–21.40)**<br>21.66 (17.03–31.77)<br>29.05 (18.86–48.04)<br>18.85 (14.25–20.06)<br>17.76 (14.37–24.96)	16.196	0.003^c^
*Multifocal tumor*<br>Unifocal tumor (n=53)<br>Multifocal tumor (n=15)	<br>17.40 (12.95–22.44)<br>27.30 (18.60–37.94)	585.00	0.006^d^

Except for the subcategories of the grade group (p=0.048), there was no statistically significant difference between the subgroups of the other variables in terms of CEA level (p>0.05).

**Figure 1:**
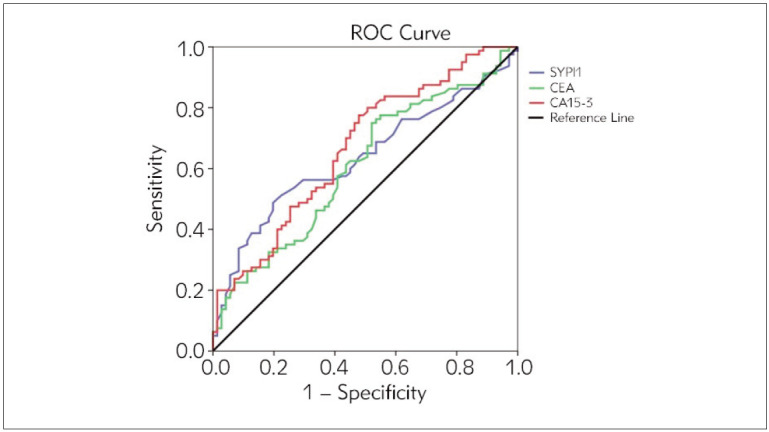
ROC curve of SYPL1, CEA, CA15-3.

Diagnostic values of SYPL1 and other variables in BC are given in Table 4. The diagnostic values of SYPL1, CEA, and CA15-3 proteins in diagnosing BC were statistically significant (p=0.002, p=0.020, and p<0.001, respectively). SYPL1>12.26; CEA>1.32; CA15-3>13.99 values are risky for BC disease (Figure 1).

**Table 4:** Diagnostic values of SYPL1 and other variables in breast cancer. PPV: Positive predictive value<br>NPV: Negative predictive value<br>SEAUC: Standart error<br>AUC: Area under the ROC curve

Variables	Cut-off	AUC	Sensitivity	Specificity	PPV	NPV	p
SYPL1	>12.26	0.639±0.047	48.75 [37.4–60.2]	80.56 [69.5–88.9]	73.6 [59.7–84.7]	58.6 [48.2–68.4]	0.002
CEA	>1.32	0.607±0.048	75 [64.1–84.0]	47.89 [35.9–60.1]	61.9 [51.4–71.5]	63.0 [48.7–75.7]	0.020
CA-15-3	>13.99	0.668±0.046	80 [66.8–86.1]	49.30 [39.9–64.1]	64.6 [54.2–74.1]	67.3 [53.3–79.3]	<0.001
CA15-3+SYPL1			32.5	87.5	74.29	53.85	

## Discussion

The most important result of our study is that serum SYPL1 is higher in BC patients than in healthy individuals. The sensitivity of serum SYPL1 in diagnosing BC was 48.75%; It was revealed that the specificity was 80.56%, and there was a statistically significant correlation with BI-RADS. The specificity of serum SYPL1 was found to be higher than CA15-3, and both its sensitivity and specificity were higher than CEA. However, it was found to be unrelated to stage, TNM groups, grade, and molecular subtypes. The results of our study show that serum SYPL1 level can be used with 48.75% sensitivity and 80.56% specificity in diagnosing BC.

Screening is basically performed with mammography to detect BC at an early stage. Mammography has been shown to reduce deaths from BC by 15–20% through early screening. However, for every 1000 women screened by mammography starting from the age of 50, 2–3 deaths from BC will be prevented. It was predicted that at least one false-positive test would be detected in 200 women, 30 would be referred for biopsy, three would be diagnosed with interval cancer, and 15 would be diagnosed with BC even though it was not [15]. In addition, mammography is insufficient in diagnosing BC in dense breast structures, and additional examinations are needed. According to the data of the Dutch screening program made according to the Wolfe classification, a 41% reduction in mortality was observed through mammographic screening in women with non-dense breasts; mortality decreases by 13% in women withdense breast structure due to decreased sensitivity of mammography [16]. The current study showed no statistically significant relationship between serum SYPL1 level and breast density classification. In this respect, we can say that breast type does not affect the serum SYPL1 level. Blood biomarkers CA15-3 and CEA were taken as reference. The sensitivity of CEA was 75%, and the specificity was 47.89%. The sensitivity of SYPL1 was 48.75%, and the specificity was 80.56%. CA15-3 had the sensitivity of 80% and the specificity of 49.30%. It was determined that serum SYPL1 could statistically significantly distinguish patients with BC from healthy individuals. When CA15-3 and SYPL1 tests are used together, it has 32.5% sensitivity and 87.5% specificity.

In a retrospective study of 149,238 BC patients in Korea, the high CA15-3 group was found at a more advanced stage compared to the normal group (Stage 3, 12.5% vs 36.1%, p<0.001) [17]. In addition, a meta-analysis of 1179 patients and 494 controls found that serum CA15-3 was closely associated with tumor stage [18]. The current study found a difference between subcategories of metastasis, stage, prognostic stage, hormone type, and multifocal tumor (multicentric-multifocal) groups in terms of CA15-3. CA15-3 value was significantly higher in patients with distant metastases, locally advancedmetastatic stages, and multifocal patients. CEA was not associated with a stage in our study, but CEA was significantly higher in grade 3 tumors than in grade 2 tumors. Serum SYPL1 level was found to be unrelated to stage and grade.

Elevated CA15-3 group was associated with ER (-), PR (-) and HER2 (+). While increased CEA is seen at a higher rate in the HER2 subtype, a lower rate in the triple-negative subtype is detected [19]. On the other hand, in the study of Li et al. [18] in 699 patients with BC under the age of 40, CEA and CA15-3 were not statistically related to the molecular subtype. In the current study, the CA15-3 value of the luminal A group was lower than the Luminal B Her2+ group. The CA15-3 value of those in prognostic stage 1B was lower than those in the prognostic stage 4. A relationship was not found between CEA and the molecular subtype.

Ki-67 level is not highly correlated with the mitotic index but is associated with mitosis [20]. As a hypothesis, the increase in mitosis in cancer cells can provide the relationship of the cancer cell with the microenvironment and increase protein synthesis. Therefore, the relationship between mitosis-related Ki-67 value and serum SYPL1 was evaluated but not statistically significant.

The diagnostic value [AUC] of developmental endothelial locus-1 (Del-1) protein in BC was 0.961 (95% CI, 0.924–0.983); its sensitivity is 94.70%; its specificity was found to be 94.70% [21]. However, it is similar to SYPL1 as a membrane protein. The involvement of SYPL1 in intracellular membrane traffic and the inclusion of Del-1 in the circulation may have affected the sensitivity and specificity values. On the other hand, Liu et al. [9] reported that SYPL1 upregulation in colon cancer tissue and serum SYPL1 level in colorectal cancer patients with adenoma and healthy. They found that it was significantly higher than that (AUC: 0.94, sensitivity: 86%, specificity: 91%). In addition, a statistically significant relationship was found with lymph node invasion, and they showed that SYPL1 level decreased in patients who underwent radical surgery for colorectal cancer. In this respect, it is seen that SYPL1 is also in circulation.

The current study showed a weak positive correlation between creatinine level and SYPL1. We thinkthat the serum SYPL1 level may change in the case of renal failure. In addition, SYPL1 can also be detected in urine, as revealed in the study by Prunotto et al. [22], in which they determined proteomics in human urine. It raises the question of whether it can be used as a marker in urothelial tumors because of its high staining in tissue in renal cell carcinoma. As for its detection in urine, the question is whether urine analysis shows similar results in colon cancer, showing high sensitivity and specificity with serum values [14].

First, the sample size was small, and studies investigating the associations between serum SYLP1 levels in a larger population of other cancer patients are relevant. Moreover, the patients were not followed up after discharge, and their baseline SYLP1 levels and association with other tumor biomarkers were not investigated.

The serum SYPL1 maintained a higher discriminatory ability for BC. The serum SYPL1 level can be used with high specificity in diagnosing BC. However, due to its low sensitivity, SYPL1 alone is insufficient for diagnosing breast cancer. Limitations of our study include the use of small patient groups and incomplete evaluation of pathological SYPL1 values. However, one of the strengths of our study is that, to the best of our knowledge, the serum SYPL1 protein has not been previously investigated in breast cancer patients. We think there is a need to study in larger patient groups by considering different parameters to elucidate the underlying mechanisms of the elevation in serum SYPL1 level.

## Dodatak

### Acknowledgment

This study was supported by The Research Fund of Istanbul University (Project No. BAP, 2021-35707).

### Author contributions statement

YOTK, OS, and HU wrote the main manuscript text, and MV, PCK, and SD prepared tables and figures. All authors reviewed the manuscript.

### Data availability statement

The datasets used and/or analyzed during the current study are available from the corresponding author upon reasonable request.

### Funding

This study was supported by The Research Fund of Istanbul University (Project No. BAP, 2021-35707).

ORCID: Hafize Uzun https://orcid.org/0000-0002-1347-8498 .

### Conflict of interest statement

All the authors declare that they have no conflict of interest in this work.
